# Parental Age of Onset of Cardiovascular Disease as a Predictor for Offspring Age of Onset of Cardiovascular Disease

**DOI:** 10.1371/journal.pone.0163334

**Published:** 2016-12-21

**Authors:** Shannon Anjelica Allport, Ngum Kikah, Nessim Abu Saif, Fonkem Ekokobe, Folefac D. Atem

**Affiliations:** 1 Perelman School of Medicine, University of Pennsylvania, Philadelphia, Pennsylvania, United States of America; 2 The University of Texas Health Science Center School of Public Health, Dallas, Texas, United States of America; 3 Trinity School of Medicine, Kingstown, St. Vincent and the Grenadines; 4 Texas A &M Health Science Center School of Medicine, Dallas, Texas, United States of America; University of Tampere, FINLAND

## Abstract

**Objective:**

The risk for cardiovascular disease (CVD) is higher for individuals with a first-degree relative who developed premature CVD (with a threshold at age 55 years for a male or 65 years for a female). However, little is known about the effect that each unit increase or decrease of maternal or paternal age of onset of CVD has on offspring age of onset of CVD. We hypothesized that there is an association between maternal and paternal age of onset of CVD and offspring age of onset of CVD.

**Methods:**

We used the Framingham Heart Study database and performed conditional imputation for CVD-censored parental age (i.e. parents that didn’t experience onset of CVD) and Cox proportional regression analysis, with offspring’s age of onset of CVD as the dependent variable and parental age of onset of CVD as the primary predictor. Modifiable risk factors in offspring, such as cigarette smoking, body mass index (BMI), diabetes mellitus, systolic blood pressure (SBP), high-density lipoprotein (HDL) level, and low-density lipoprotein (LDL) level, were controlled for. Separate analyses were performed for the association between maternal age of onset of CVD and offspring age of onset of CVD and the association between paternal age of onset of CVD and offspring age of onset of CVD.

**Results:**

Parental age of onset of CVD was predictive of offspring age of onset of CVD for maternal age of onset of CVD (P < .0001; N = 1401) and for paternal age of onset of CVD (P = 0.0134; N = 1221). A negative estimate of the coefficient of interest signifies that late onset of cardiovascular events in parents is protective of onset of CVD in offspring. Cigarette smoking and HDL level were important associated confounders.

**Conclusions:**

Offspring age of onset of cardiovascular disease is significantly associated with both maternal and paternal age of onset CVD. The incorporation of the parameters, maternal or paternal age of onset of CVD, into risk estimate calculators may improve accuracy of identification of high-risk patients in clinical settings.

## Introduction

Cardiovascular disease (CVD), defined as coronary death, myocardial infarction, coronary insufficiency, angina, ischemic stroke, hemorrhagic stroke, transient ischemic attack, peripheral artery disease, and heart failure, is the leading global cause of death, accounting for over 30 percent of all deaths worldwide– 17.3 million deaths per year. In the United States, someone dies from cardiovascular diseases every 39 seconds [1]. Though the death rate due to CVD has decreased slowly over the last 5 decades, heart disease remains the leading cause of death in the United States, and caring for patients with poor cardiovascular health continues to be one of the largest burdens on the health care system today. From 1990 to 2009, CVD ranked first in the number of days for which patients received hospital care [2], yet 72% of Americans do not consider themselves at risk for heart disease [1].

Associations have long been established between CVD and a wide variety of risk factors, including non-modifiable variables such as age, sex, and family history, and modifiable atherosclerotic risk factors, such as cigarette smoking, alcohol intake, increased BMI, systolic blood pressure (SBP), diabetes mellitus, high-density lipoprotein (HDL) level, and hypercholesterolemia [3–10]. High prevalence of CVD within a family can indicate shared environmental, cultural, and behavioral factors, and presence of these aforementioned risk factors were previously thought to explain much of the aggregation of CVD in certain families. For example, shared environmental risk factors, such as the presence of smoke and unhealthful diet, accounts for some of the increased risk of CVD in offspring with affected parents. However, the INTERHEART study established that parental history of coronary heart disease confers increased risk for the development of myocardial infarction (MI), independent of other established risk factors [11]. Additionally, genetic markers involved in lipoprotein handling, endothelial integrity, arterial inflammation, and thrombosis formation have been linked to increased risk of CVD in families [12, 13].

Despite this evidence that recognizes that both parental history and genetic factors are modifiers of MI risk, neither element is routinely used in the clinical determination of CVD risk. Currently, few analyses delineate the association between parental history of CVD and manifestations of CVD other than MI (subclinical atherosclerosis, unstable angina, and stable angina). Furthermore, measuring parental history as a binary variable (“yes” or “no”) is an oversimplification that fails to recognize the effects of several variables within the parental history, including the relatives’ age of onset of disease, patient’s relation to relative, and quantity of relatives affected with cardiovascular disease, all of which have been shown to impact CVD risk [14–17].

The Framingham Heart Study has published multivariate models for estimation of the 10-year absolute risk of developing CVD [18,19], and the European SCORE project developed a similar coronary risk-assessment calculator [20]. These multivariate equations and risk scores are used to develop clinical guidelines that dictate whether patients are appropriate candidates for statin drug therapy for primary prevention of CVD [21,22]. However, these risk-assessment tools fail to include positive parental history as a determinant of CVD risk. As the Framingham risk-score underestimates subclinical atherosclerosis in certain populations, including women and persons of low socioeconomic status [23,24], better characterization and application of parental history may improve the accuracy of this tool. There is a need to improve these calculators to aid in clinical decision-making, particularly for patients with a parental history of CVD who are determined to be at intermediate risk with moderate environmental risk factors who do not have current indications for prophylactic therapy [25]. Characterization of the effect of parental age of CVD onset on offspring CVD risk will aid in determining true risk and help to guide decisions on whether to start prophylactic treatment, when treatment should start, and how aggressively to treat other modifiable risk factors.

While the effect of parental history of premature cardiac events (<55 years old in men, <65 in women) and the effects of advanced age on CVD risk is well-studied [26,27], no research has been done on assessing the risk of a unit increase or decrease of parental age of onset of CVD and offspring age of onset of CVD while controlling for modifiable risk factors. Establishing this relationship on a continuous scale as compared to thresholding at age 55 years and 65 years will lead to a more powerful test and a better prediction of offspring age of CVD onset. Furthermore, past studies oversimplify the parental history to a dichotomous variable. The objective of this investigation is to evaluate the relationship between age of CVD onset in parents and age of CVD onset in offspring while accounting for the effect of each unit increase or decrease on the hazard ratio for cardiovascular events in offspring. Delineation of this relationship may identify a population that would benefit from more aggressive risk factor modification for primary prevention and who would otherwise have not been treated using current clinical calculators for CVD risk.

## Materials and Methods

The study was approved by University of Texas Health Science Center in Houston Institutional Review Board and Biologic Specimen and Data Repository Information (BioLINCC). For this study we used The Framingham Heart Study (FHS) database. The data comes from a well-known, longitudinal prospective cohort study that is a premier database for study of cardiovascular diseases and other terminal disease. The FHS Original Cohort was launched at Exam 1 in 1948 and has continued with biennial examinations to the present. The FHS Original Cohort consists of 5,209 respondents (55% females) aged 28–62 years residing in Framingham, Massachusetts, between 1948 and 1951. Nearly all subjects were Caucasians. The Offspring Cohort (FHSO) was launched at Exam 1 in 1971 and has on average been examined every 3 to 4 years since enrollment. The FHSO dataset consists of a sample of 3514 biological descendants of the Original Cohort, 1576 of their spouses and 34 adopted offspring for a total sample of 5124 subjects (52% females). This analysis used values collected during the FHS Original Cohort Exam 12 (1971–1974, *n* = 3261) and the FHSO Exam 1 (1971–1975, *n* = 5124). There was a total of 2622 linked parent-offspring pairs. The age of onset of CVD is the age at which either parent or offspring was diagnosed with CVD, including coronary death, myocardial infarction, coronary insufficiency, angina, ischemic stroke, hemorrhagic stroke, transient ischemic attack, peripheral artery disease, and heart failure, by a medically trained professional. This ‘time-to-event’ data was collected based on follow up data of both the original and offspring cohorts. Baseline values of the covariates of interest, collected at first clinical visit, were used in data analysis. Participants were included if they had not yet developed overt symptoms of cardiovascular disease or suffered a heart attack or stroke, and subjects were excluded if they had missing data.

The research protocols of the Framingham Heart Study are reviewed annually by the Institutional Review Board of the Boston University Medical Center and by the Observational Studies Monitoring Board of the National Heart, Lung and Blood Institute. Since 1971, written consent has been obtained from participants before each examination. The deterministic linkage of the two datasets was possible, thanks to the Framingham Executive Committee that provided anonymous patient ID and a specific family code.

In our study, in order to evaluate the relationship between age of CVD onset in parents and age of CVD onset in offspring, we controlled for offspring modifiable risk factors such as cigarette smoking, diabetes mellitus, BMI, SBP, HDL, and LDL. Similar to the thresholding approach that allocates different risk for male and female first-degree relatives, separate analyses were performed for each parent.

## Statistical Analysis

We used Cox’s proportional hazards model [28] to examine the relationship between offspring age of onset of CVD and parent age of onset of CVD. As an illustration for our paper, we are interested in estimating the parameters of the Cox proportional hazards model of the relationship between parent age of onset of cardiovascular disease as a primary predictor while controlling for offspring baseline cigarette smoking status, diabetes mellitus, BMI, SBP, HDL, and LDL. We used the time of onset of cardiovascular disease in offspring participants as the outcome of interest. The primary variables of interest, paternal and maternal age of onset of CVD, were imputed using the single imputation for a randomly censored covariate. Potential confounding variables (BMI, SBP, HDL, and LDL) were entered as continuous variables while diabetes status and smoking status were entered as categorical variables. Non-diabetics and non-smokers, respectively, were the designated reference groups.

SAS software (Version 9.4) was used for statistical analysis. The proportional hazard regression calibration approach employed in this paper for analyzing the time to event outcome and censored predictor is similar to the proportional hazards regression model for survival data with measurement error [29,30]. This Cox regression calibration approach for missing data by Prentice [30] was extended to accommodate the association of a random censored predictor and a random censored dependent variable. The conditional expectation approach presented in this paper closely resembles the regression method that has been applied and investigated for missing covariates in a variety of regression models, including linear regression models presented by Huang (2005) [31], the Cox regression model [29,30,32,33], and the logistic regression model [34–36]. Statistical justification for the robust approach employed in this paper is provided by Dupuy and Leconte (2009) [33] for missing covariates with survival outcomes.

Unlike binary thresholding approaches that have shown increased risk of CVD if a first-degree blood relative has had coronary heart disease or a stroke before the age of 55 years (for a male) or 65 years (for a female), we can compute a hazard ratio for each unit increase or decrease of parent age of onset. The conditional imputation approach employed in this paper combined the non-modifiable risk factors, age of onset of CVD in mother or father and parental history, by defining parental onset of CVD as the instantaneous risk of cardiovascular event, given that the event has not previously occurred. This leads to a relatively stable estimate and avoids bias estimates as the thresholding approach shown by Rigobon & Stocker [37] and Austin & Hoch [38] have. Similar to the thresholding approach, our approach also controls for modifiable risk factors such as cigarette smoking, diabetes mellitus, BMI, SBP, HDL, and LDL. Basic statistics were preformed to describe the distribution of frequency, mean value, and range of the confounding variables (Tables 1 and 2).

**Table 1:** Baseline characteristics of offspring based on maternal and offspring associations.

Variable		Mean (SD)	N (%)
**CVD Offspring**	No Event		1013 (72.31)
	Event		388 (27.69)
**CVD in Mother**	No Event		907 (64.74)
	Event		494 (35.26)
**Smoke**	No		761 (54.32)
	Yes		640 (45.68)
**Diabetes**	No		1316 (98.58)
	Yes		19 (1.420)
**BMI**		25.77 (6.46)	
**SBP**		129.03 (17.19)	
**HDL**		50.67 (14.64)	
**LDL**		124.23 (34.84)	

**Table 2:** Baseline characteristics of offspring based on paternal and offspring associations.

Variable		Mean (SD)	N (%)
**CVD in Offspring**	No Event		938 (76.82)
	Event		283 (23.18)
**CVD in Father**	No Event		909 (74.45)
	Event		312 (25.55)
**Smoke**	No		707 (57.90)
	Yes		514 (42.10)
**Diabetes**	No		1154 (98.63)
	Yes		16 (1.370)
**BMI**		25.36 (4.390)	
**SBP**		127.96 (17.27)	
**HDL**		50.69 (14.21)	
**LDL**		119.72 (33.29)	

The age of onset of CVD is defined as the age at which either parent or offspring was diagnosed with CVD by a medically trained professional. The dependent variable, age of onset of CVD in offspring, is potentially right-censored. That is, offspring that truly had CVD but were not diagnosed with CVD by a medical professional during this study are considered censored. Similarly, for the primary predictor variable, age of onset of CVD in parents, those that truly had CVD but were not diagnosed with CVD are considered censored. In order to consider`censored parents in the Cox model for age of onset of CVD in offspring, we employed the conditional mean imputation technique [37,38].

## Results

Our primary variable of interest is parental age of onset of CVD, which is potentially right-censored. We controlled for the following confounders: offspring smoking status, offspring diabetes status, offspring BMI, offspring SBP, offspring HDL, and offspring LDL (Tables 3 and 4). The outcome, offspring age of onset of CVD, is survival data, data where the outcome variable is the time until the occurrence of an event of interest. The offspring age of onset is survival data because the variable is recorded as the time from birth to the development of CVD. In this cohort, not all of the offspring developed CVD. The age at which they were diagnosed with CVD by a physician was recorded as ‘age of onset’ while those who did not develop CVD were censored. The group that did not develop CVS is considered censored because we assume that they have a non-zero chance of developing CVD later in life but are CVD free at the present time.

**Table 3:** Survival analysis for the association between maternal age at onset of CVD and offspring age at onset of CVD.

Parameter	Estimate (SE)	Hazard Ratio	p-value
**Maternal Age of onset of CVD**	-0.0423 (0.0096)	0.959	< .0001*
**Smoke**	0.5628 (0.1563)	1.756	0.0003*
**BMI**	0.5857 (0.5282)	1.796	0.2674
**Diabetes**	-0.8187 (1.0310)	0.441	0.4271
**SBP**	-0.0059 (0.0043)	0.994	0.1742
**HDL**	-0.9053 (0.2799)	0.404	0.0012*
**LDL**	-0.0037 (0.0022)	0.996	0.0933

**Table 4:** Survival analysis for the association between paternal age at onset of CVD and offspring age at onset of CVD.

Parameter	Estimate (SE)	Hazard Ratio	p-value
**Paternal Age of onset of CVD**	-0.0270 (0.0109)	0.973	0.0134*
**Smoke**	0.2989 (0.1801)	1.348	0.0970
**BMI**	0.0334 (0.5548)	1.034	0.9520
**Diabetes**	0.1902 (0.7503)	1.209	0.7999
**SBP**	-0.0098 (0.0050)	0.990	0.0503
**HDL**	-1.2687 (0.3148)	0.281	< .0001*
**LDL**	-0.0011 (0.0029)	0.999	0.7162

**Fig 1:**
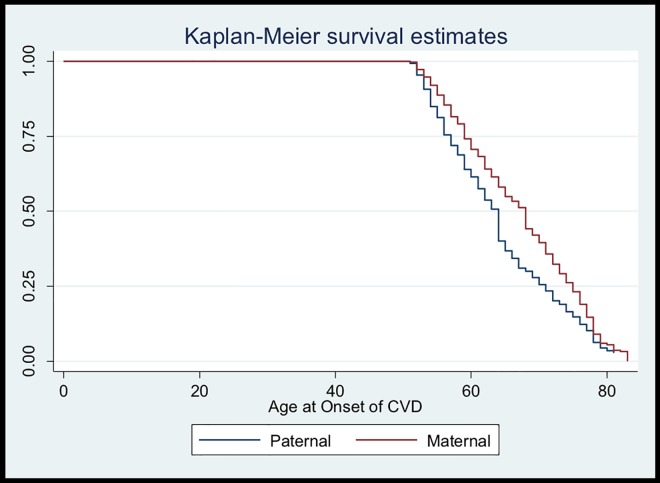
Kaplan-Meier Survival Estimates.

Separate analyses were performed for each parent, and the data was separated into two groups. There were 2622 linked parent-offspring pairs in total, with 1401 mother-offspring pairs and 1221 father-offspring pairs. The median age of onset of CVD was 68 years and 64 years for mothers and fathers, respectively (Fig 1). The overall median age of onset of CVD in parents was 64 years.

The baseline characteristics for the separate analysis with the primary predictors, maternal age of onset of CVD and paternal age of onset of CVD, are shown in Table 1 and Table 2, respectively. Out of 1401 mothers, 45.68% of their offspring smoked and 1.42% were diabetic. Maternal mean(SD) BMI, SBP, HDL, and LDL values were 25.77(6.46), 129.03(17.19), 50.67(14.64), and 124.23(34.84) respectively (Table 1). Of the 1221 fathers, 42.10% of their offspring smoked and 1.37% were diabetic. Paternal mean(SD) BMI, SBP, HDL, and LDL values were 25.36(4.39), 127.96(17.27), 50.69(14.21), and 119.72(33.29), respectively (Table 2).

**Fig 2:**
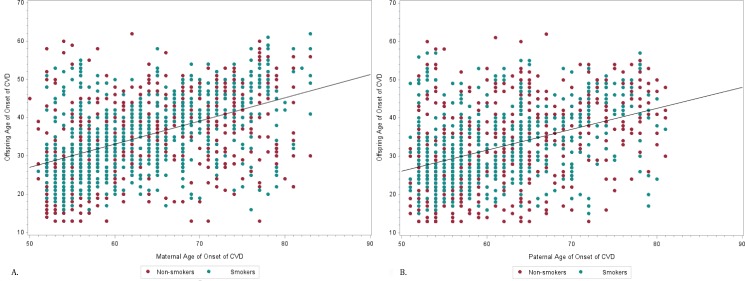
A, Correlation of maternal age of onset of CVD and offspring age at onset of CVD with fixed smoking status. B, Correlation of paternal age of onset of CVD and offspring age at onset of CVD with fixed smoking status.

The Cox proportional hazard (Cox PH) models were used to analyze the relationship between offspring age of onset of CVD and parental age of onset of CVD. We presented a separate analysis for the association between offspring age of onset of CVD and maternal age of onset of CVD and offspring age of onset of CVD and paternal age of onset of CVD (Tables 3 and 4). The primary variables of interest, maternal and paternal age of onset of CVD, are statistically significantly related to age of onset of CVD in offspring with hazard ratios of 0.959 and 0.973, respectively. The negative estimates of the coefficients of interest, maternal and paternal age of onset of CVD, are -0.0423 and -0.0109, respectively. This signifies that late onset of cardiovascular events in mothers and fathers is protective of onset of CVD in offspring. The most important confounders for offspring age of onset of CVD in the maternal analysis were smoking (P = 0.0003) and HDL (P = 0.0012), and, for the paternal analysis, HDL (< .0001). Fig 2A and 2B show plots offspring age at onset of CVD against maternal/paternal age at onset of CVD, with an important confounder, smoking, fixed and separated. These plots illustrate that while there is a weak pattern for non-smokers, smokers tend to have a stronger correlation between parental and offspring onset of CVD.

**Fig 3:**
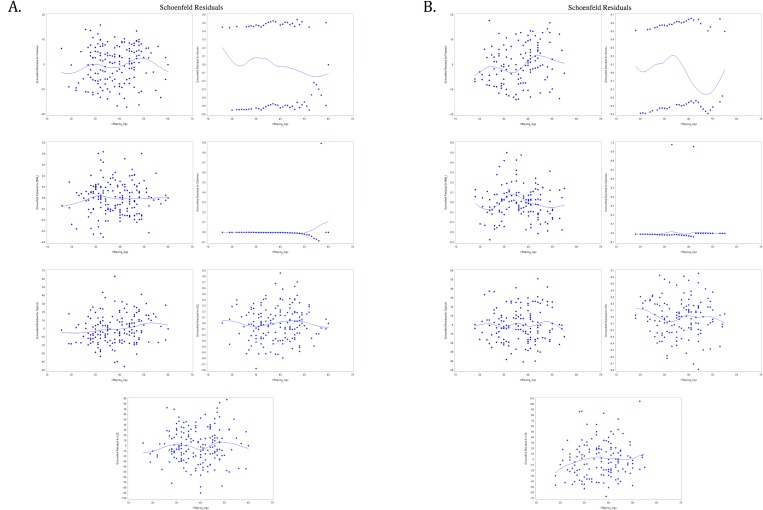
A, Schoenfeld Residual for offspring age of onset and variables maternal age of CVD onset, smoking status, BMI, Diabetes, SBP, HDL and LDL, respectively. B, Schoenfeld Residual for offspring age of onset and variables paternal age of CVD onset, smoking status, BMI, Diabetes, SBP, HDL and LDL, respectively.

We fit a Cox model for the association of age of onset of CVD in parents and offspring. The Schoenfeld residuals (Schoenfeld, 1982) against age of onset CVD in offspring with a smooth line (cubic spline) are fit to the points (Fig 3A and 3B). These smooth lines help to visualize the plots, and show that there is no clear pattern with offspring age of CVD onset, i.e. there is no indication of a lack of fit of the model to individual observations.

## Discussion

Maternal age of onset of CVD is statistically significantly related to age of onset of CVD in offspring with a hazard ratio of 0.959, while paternal age of onset has a hazard ratio of 0.973. Our analysis describes hazard ratios based on unit increase or decrease of maternal and paternal age of onset of cardiovascular events. Although several papers have evaluated the link between CVD in parents and CVD risk in offspring, they have categorized parental history of CVD as a dichotomous variable using a thresholding approach [26,39] or in 10-year intervals [40], which decreases the power of the analysis. None have shown the effect of specific age of onset of CVD in parents on the risk of CVD in offspring. Our results are in line with previous knowledge that late onset CVD decreases hazard ratio and is protective of CVD development in offspring [26,27], but also increases power by providing a greater level of detail of this relationship.

This is the first study to link parental age of onset to offspring age of onset of CVD using continuous variables in survival data. Currently, multivariate models for estimation of the 10-year absolute risk of developing CVD, including the Framingham calculator and the European SCORE calculator, can underestimate subclinical atherosclerosis [23,24]. Neither calculator uses parental history as a variable in the calculation of 10-year cardiovascular disease risk, but acknowledge that risk may be higher than indicated in the calculators for those with a “strong family history of premature CVD” [41]. Basing both the dependent variable, offspring age of onset of CVD, and the independent variable, fathers’ and mothers’ age of onset of CVD, on unit increase or decrease, as opposed to threshold, allows for a more precise estimate of risk in each unique circumstance. This method also allows us to develop age-specific hazard ratios for onset of cardiovascular disease. A benefit of using a continuous variable for age of CVD onset in calculation of CVD risk is the improved accuracy of identification of high-risk patients that could benefit from prophylactic therapy and more aggressive treatment of other CVD risk factors.

Several studies have suggested that a maternal transmission of CVD is more important for the development of CVD than paternal transmission [42–44]. Some studies have not found statistically significant differences between the effect of maternal and paternal CVD on offspring CVD [45,46], while even fewer have concluded that paternal history of CVD conferred a higher risk of the disease [47]. In our study, both hazard ratios for the effect of maternal and paternal age of onset of CVD were statistically significant. A meta-analysis of 26 cohort and cross-sectional studies concluded that the conferred risk of CVD in offspring was not substantially different between positive paternal and maternal histories of CVD [48]. Since a positive family history confers increased risk of CVD, presence of parental history and ages at onset are useful for determination of risk, but the utility of making a distinction between whether the mother or father was affected is not currently clear.

The Framingham data comes from a well-known prospective cohort study. Advantages of using the Framingham Heart Study data include access to near-complete data on the development of selected cardiovascular diseases due to their careful longitudinal follow-up of subjects. The Framingham Study was the first large scale cohort study of its kind that compare one generation to the next using a wide variety of data parameters. However, practitioner error may lead to a CVD sensitivity and specificity of less than 100%. That is, some subjects with CVD may be diagnosed an indefinite period of time after time of actual CVD onset or not at all, introducing information bias into the study. As technologies for detection of CVD (including vascular CT arteriograms, microalbuminuria testing, and optic fundoscopy, to name a few) have improved dramatically, CVD can be detected more definitively at an earlier time in the disease course and subsequent cohorts (offspring) may have experienced different sensitivity and specificity of diagnosis. Furthermore, nearly all subjects enlisted in the study in 1948 were White. The homogeneity of the cohorts may limit generalizability to a more diverse population, and future studies should include subjects from a variety of ethnic backgrounds to improve the ability to apply findings to the general population.

Lastly, the statistical interpretation of this study can be confusing; both independent and dependent variables are potentially right-censored so they may not have clean interpretations. However, based on the sign of the coefficient (negative) it can be determined that the increasing parental age of onset of CVD is protective of early CVD in offspring.

Further research on the role of family history of CVD, including age of onset, number of family members affected, and differential effect of sex of family members affected, and its implications for CVD risk should be explored. The Framingham data is an excellent body of data for use in analyses of this nature, and future research should focus on risk factor studies in the 3^rd^ and 4^th^ generation of this cohort study. More comprehensive understanding of cardiovascular risk factors will allow practitioners to more accurately identify patients at high risk for CVD who would not otherwise be identified by traditional risk calculators.
